# The Influence of Light Irradiation on the Photocatalytic Degradation of Organic Pollutants

**DOI:** 10.3390/ma13112494

**Published:** 2020-05-29

**Authors:** Alexandru Enesca, Luminita Isac

**Affiliations:** Product Design, Mechatronics and Environmental Department, Transilvania University of Brasov, Eroilor 29 Street, 35000 Brasov, Romania; isac.luminita@unitbv.ro

**Keywords:** TiO_2_, doctor blade, photocatalysis, light spectra, photon flux, kinetics

## Abstract

The design of a photocatalytic process must consider intrinsic and extrinsic parameters affecting its overall efficiency. This study aims to outline the importance of balancing several factors, such as radiation source, total irradiance, photon flux, catalyst substrate, and pollutant type in order to optimize the photocatalytic efficiency. Titanium oxide was deposed by the doctor blade technique on three substrates (microscopic glass (G), flour-doped tin oxide (FTO), and aluminum (Al)), and the photocatalytic properties of the samples were tested on two pollutants (tartrazine (Tr) and acetamiprid (Apd)). Seven irradiation scenarios were tested using different ratios of UV-A, UV-B + C, and Vis radiations. The results indicated that the presence of a conductive substrate and a suitable ratio of UV-A and Vis radiations could increase the photocatalytic efficiency of the samples. Higher efficiencies were obtained for the sample Ti_FTO (58.3% for Tr and 70.8% for Apd) and the sample Ti_Al (63.8% for Tr and 82.3% for Apd) using a mixture of three UV-A and one Vis sources (13.5 W/m^2^ and 41.85 μmol/(m^2^·s)). A kinetic evaluation revealed two different mechanisms of reaction: (a) a one-interval mechanism related to Apd removal by Ti_FTO, Ti_Al (scenarios 1, 4, 5, and 7), and Ti_G samples (scenario 7) and (b) a two-interval mechanism in all other cases.

## 1. Introduction

Sustainability is a key parameter to be considered when designing environmental treatment processes for pollutant removal from wastewater, air, or soil. The light absorption range is an important factor that directly influences the photocatalytic activity of a semiconductor. Therefore, the influence of the irradiation light on the photocatalytic performance of various semiconductors must be studied because it can provide a basis for a further understanding of the photocatalysis principles. Additionally, there must be a balance between energy consumption and photocatalytic efficiency in order to properly design a photocatalytic technology [1,2,3]. 

Advanced oxidation processes (AOPs) based on semiconductor photocatalysts are recognized to remove, through mineralization, organic pollutants in low concentrations, including emergent organic dyes [4,5], pesticides [6,7], pharmaceutical compounds [8,9], etc. The economic factor is usually considered very relevant, as AOPs are energy-intensive [10,11], especially due to the most commonly employed UV activation [12,13,14]. Until now, many papers have reported the use of AOPs especially based on composites [15,16], semiconductors [17,18,19], or hybrid systems [20,21]. It is known that photocatalysis aims at the use of light radiation, with infield radiation values of hundreds of W/m^2^. Based on practical reasons, most laboratory-scale experiments are developed at lower or even different irradiance values. Consequently, it is difficult to compare results when the kinetics of the photocatalytic process is influenced by the input radiation.

This paper presents several parameters that should be considered when designing a photocatalytic experiment: irradiation sources, total irradiance, photon flux, catalyst substrate, and pollutant type. Three types of substrate were used for TiO_2_ deposition by the doctor blade technique: microscopic glass (G), flour-doped tin oxide (FTO), and aluminum. During the photocatalytic experiments, the influence of UV-A, UV-B + C, and Vis radiations (in various ratios) was investigated for two types of pollutants: a dye (tartrazine) and a pesticide (acetamiprid). The study includes an evaluation of the kinetic mechanism based on the influence of the photon flux corresponding to each irradiation scenarios.

## 2. Materials and Methods 

### 2.1. Photocatalyst Materials

Three samples were prepared based on TiO_2_ Degussa P25 powder as follows:

1. Sample Ti_G: a TiO_2_ film was deposed on a microscopic glass substrate by the doctor blade technique. Previously, the substrate was degreased with surfactants and cleaned by successive immersion in acetone and ethanol for 15 min using an ultrasound bath. After deposition, the samples where thermally treated at 500 °C for 6 h.
2. Sample Ti_FTO: a TiO_2_ film was deposed on a flour-doped tin oxide substrate by the doctor blade technique; similar cleaning and thermal procedures as for Ti_G were used.
3. Sample Ti_Al: a TiO_2_ film was deposed on an aluminum substrate by the doctor blade technique.

Previously, the substrate was degreased with surfactants, cleaned with ethanol, polished, and dried at room temperature. A thin layer of Al_2_O_3_ was developed on the surface of the aluminum substrate using an electrochemical setup consisting of a platinum cathode, an aluminum anode, and 25% H_2_SO_4_ as the electrolyte. The technological parameters were voltage 12.6 V, current 1 A/dm^2^ for 30 min. The samples were cleaned again in ethanol after the electrochemical process. The post-deposition thermal treatment was done at 200 °C for 6 h.

A paste was obtained by dispersing 0.5 g TiO_2_ Degussa P25 powder into solutions containing ethanol, acetylacetonate, and Triton X100 in a volumetric ratio of 10:1:1. The samples’ size was identical (1.5 × 2.5 cm^2^), and the same deposition procedure was used to obtain the TiO_2_ film. 

### 2.2. Photocatalytic Experiments

A photocatalytic reactor was designed to accomplish several conditions: (1) uniform light intensity distribution using 4 light sources, (2) low-humidity environment, and (3) stable environmental temperature (between 20 and 25 °C). Several light scenarios where used as presented in Table 1. The UV irradiation sources were black light tubes (F18W/T8, UVA, typically 340–400 nm, with λ_UVA,max_ = 365 nm, flux intensity 3Lx, Philips, New York, NY, USA), fluorescent tubes (T38 TL 20W/12, UVB + C, typically 210–310 nm, with λ_UVB + C,max_ = 295 nm, flux intensity 2.4Lx, Philips, New York, NY, USA), and Vis white cold light tubes (TL-D Super 80 18W/865, typically 400–700 nm, with λ_Vis,max_ = 565 nm, flux intensity 28Lx, Philips, New York, NY, USA).

Each irradiation scenario was applied to all samples using two reference pollutants, i.e., the dye tartrazine (Tr) and the pesticide acetamiprid (Apd). Each sample was immersed in 30 mL of pollutant solution (0.025 mM) for 10 h. In the first 2 h, the samples were kept in the dark in order to reach absorption equilibrium. During the following 8 h, the samples were irradiated using one of the scenarios presented in Table 1. The variation in concentration was evaluated based on the UV–Vis calibration curve and hourly measured, up to 8 h of photo-catalysis. Consequently, of the total experiment period of 10 h, 2 h where without irradiation and 8 h with irradiation.

The photocatalytic efficiency was evaluated using Equation (1):

(1) $$\eta =\left[\frac{\left(C_{0}-C\right)}{C_{0}}\right]\times 100,$$

where *C*_0_ represents the initial concentration, and C represents the pollutant concentration at moment t.

### 2.3. Investigation Instruments

The crystalline structure was investigated using X-ray diffraction (XRD, Bruker D8 Discover Diffractometer (Karlsruhe, Germany), the locked-couple technique with 0.002-degree scan step and 0.01 s/step). The samples’ morphology was evaluated using scanning electron microscopy (SEM, Hitachi model S–3400 N type 121 II, Fukuoka, Japan) in a high vacuum regime. A UV–Vis spectrometer (Perkin Elmer Lambda 950, Waltham, MA, USA) was used to monitor the photocatalytic activity and to evaluate the photocatalytic kinetics. Total irradiance values were measured in the central position of the sample holder, using a class A pyranometer (SR11, Hukseflux, Berlin, Germany).

## 3. Results and Discussions

### 3.1. Crystalline Structure and Morphology

**Figure 1:**
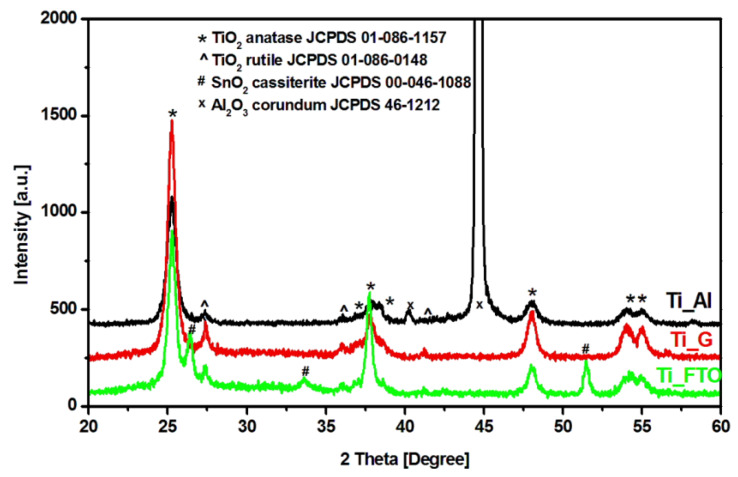
X-Ray diffraction pattern corresponding to Ti_Al (TiO_2_ film on an aluminum substrate), Ti_G (TiO_2_ film on a microscopic glass substrate), and Ti_FTO (TiO_2_ film on a flour-doped tin oxide substrate) samples.

The presence of a crystalline structure is considered as prerequisite in photocatalytic applications due to the importance of charge carriers (electrons and holes) for the development of oxidative species. It is well known that TiO_2_ anatase is the most photoactive structure in the photocatalytic degradation of organic pollutants [22,23]. However, Scanlon et al. [24] showed that using both rutile and anatas crystalline, TiO_2_ structures will promote photon conversion based on the effective band gap shift toward Vis radiation (up to 2.81 eV). Diffraction analysis (see Figure 1) showed that all samples contained both anatase and rutile TiO_2_ structures, and no additional peaks corresponding to other TiO_2_ structures were detected. The sample Ti_FTO exhibited SnO_2_ tetragonal structure peaks characteristic of the conductive FTO layer, which could interfere with the charge carrier mobility during the photocatalytic activity. Additional Al_2_O_3_ peaks were present for the Ti_Al sample as a consequence of the anodization process.

**Figure 2:**
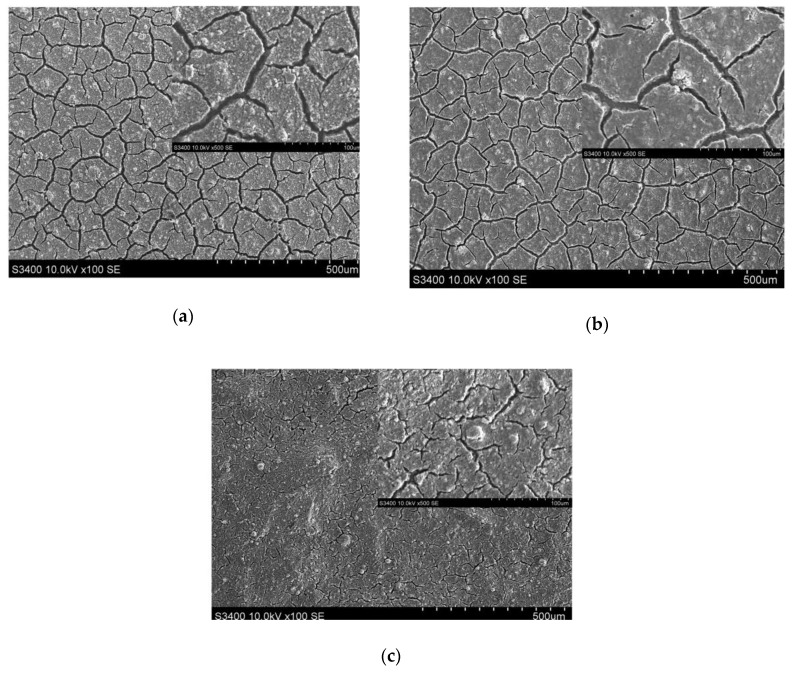
SEM images corresponding to (**a**) Ti_G sample annealed at 500 °C; (**b**) Ti_FTO sample annealed at 500 °C, and (**c**) Ti_Al sample annealed at 200 °C (inset shows SEM images at higher resolution corresponding to the same samples in the respective images).

The morphology was investigated by scanning electron microscopy in semi-vacuum without metallic coverage, and the results are presented in Figure 2. Due to the doctor blade deposition process characteristics which induce the formation of thick layers and the high-temperature thermal treatment required to eliminate the organic materials, the samples presented large uniformly distributed fractures. Several papers [25,26,27] have shown that these fractures can act as high-energy active sites during the photocatalytic activity, which is an interface-dependent process. Smaller fractures were present in the Ti_Al sample, for which the thermal treatment was kept at 200 °C , a temperature significantly lower compared to that of the other two samples (500 °C as annealing temperature).

### 3.2. Photocatalytic Properties

**Figure 3:**
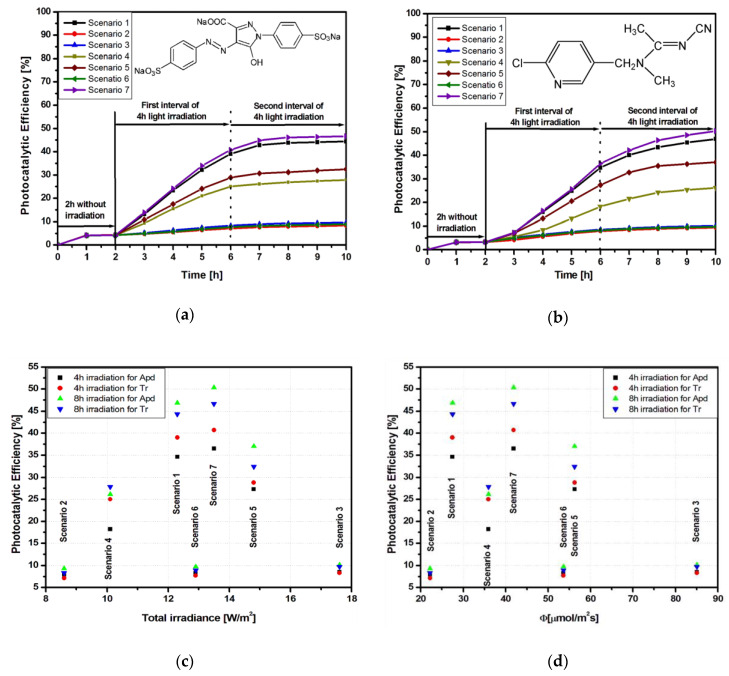
Photocatalytic parameters of the Ti_G sample: (**a**) tartrazine (Tr) photodegradation; (**b**) acetamiprid (Apd) photodegradation; (**c**) total irradiance vs. photocatalytic efficiency, and (**d**) photon flux vs. photocatalytic efficiency.

The influence of the different radiation schemes on the photocatalytic properties of the Ti_G sample regarding the degradation of Tr and Apd are presented in Figure 3a,b. Higher photocatalytic efficiency was observed for Apd in comparison to Tr. When Tr was used as a pollutant molecule, the available photon flux to the photocatalyst surface decreased, because light penetration through dye solutions is low [28,29]. The highest efficiencies (50.3% for Apd and 46.6% for Tr) correspond to scenario 7, which used three UV-A and one Vis sources. Figure 3c shows that the photocatalytic efficiency was dependent not only on total irradiation but also on the irradiation source. Similar low efficiencies were obtained at 8.6, 12.9, and 17.6 W/m^2^, while higher efficiencies corresponded to 12.3 and 12.9 W/m^2^. These results can be explained by the conversion of the total irradiance into a photon flux that was involved in the photocatalytic processes (see Table 2).

On average, the irradiance values corresponding to one single irradiation source were: E_UVA_ = 3.1 W/m^2^ for the UV-A source(s), E_UVB+C_ = 2.15 W/m^2^ for the UV-B + C source(s), E_Vis_ = 4.4 W/m^2^ for the Vis source(s). Based on these values, on the number of sources (n_uv_ and, respectively n_vis_), and on the maximum wavelength of the sources (λ_UV, max_, λ_Vis, max_), the maximum photon flux reaching the quartz beaker during each experimental trial, Φ, was calculated using Equation (2), [30]:

(2) $$\Phi =\frac{E_{\mathrm{UV}}\times \lambda_{\mathrm{UV}\;}\times n_{\mathrm{UV}}+{\;E}_{\mathrm{Vis}}\times \lambda_{\mathrm{Vis}}\times n_{\mathrm{Vis}}}{h\;\times \;c\;\times {\;N}_{\mathrm{Av}}},$$

where the Planck constant (h), the speed of light (c), and the Avogadro number have the usual values.

The efficiencies for scenarios 1 and 7 were similar even if the photon flux (Figure 3d) was almost double. This is an indicator showing that increasing the photon flux is not enough if there is no correspondence with the photocatalyst effective band gap. Literature data [31,32] mention that the energy corresponding to the effective band gap is the one that has to be surpassed for photo-activation, which, in the case of Degussa TiO_2_ (containing both anatase and rutile structures), is 2.81 eV.

**Figure 4:**
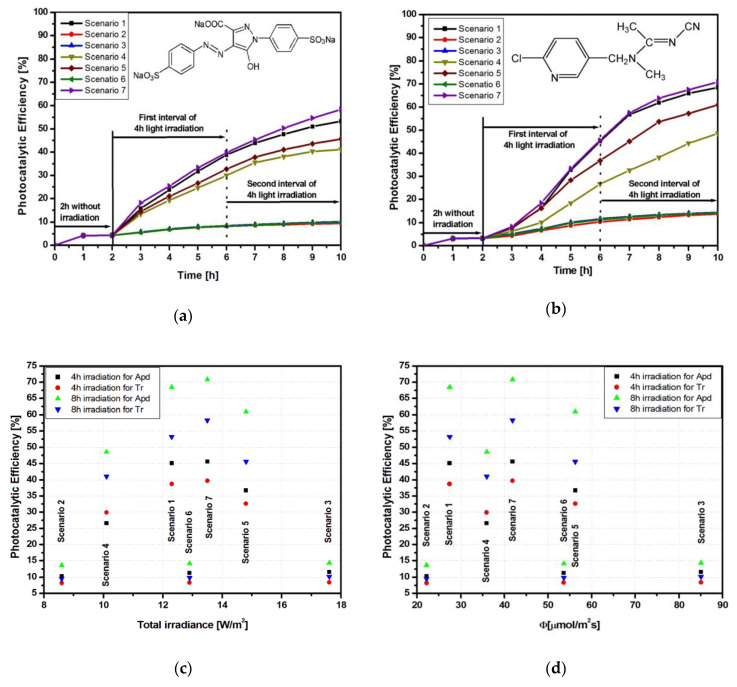
Photocatalytic parameters of the Ti_FTO sample: (**a**) Tr photodegradation, (**b**) Apd photodegradation, (**c**) total irradiance vs. photocatalytic efficiency, and (**d**) photon flux vs. photocatalytic efficiency.

**Figure 5:**
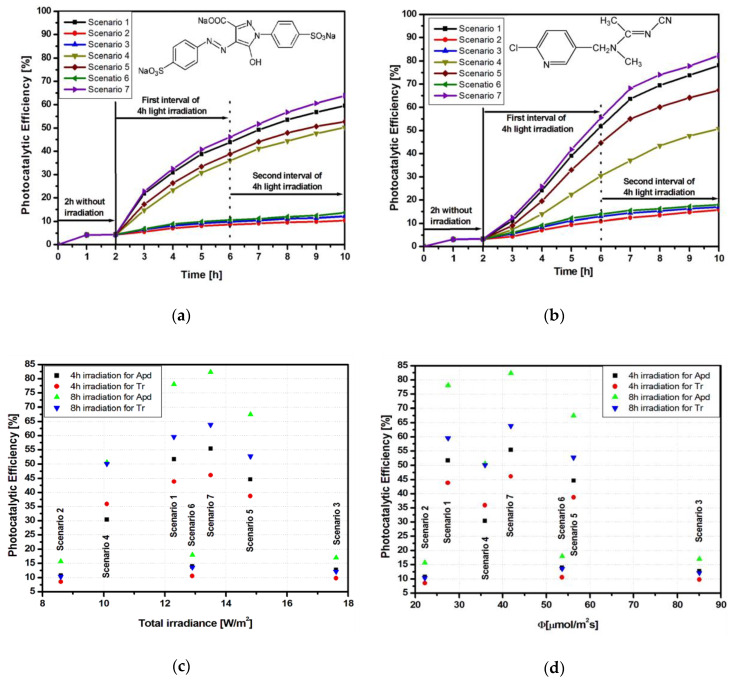
Photocatalytic parameters of the Ti_Al sample: (**a**) Tr photodegradation; (**b**) Apd photodegradation; (**c**) total irradiance vs. photocatalytic efficiency, and (**d**) photon flux vs. photocatalytic efficiency.

A similar behavior was observed for samples Ti_FTO and Ti_Al. However, in comparison with Ti_G, the other two samples showed better photocatalytic efficiency for both Tr (Figure 4a and Figure 5a) and Apd (Figure 4b and Figure 5b) pollutants.

The increase of photocatalytic efficiency for the sample Ti_FTO (58.3% for Tr and 70.8% for Apd) is related to the light conversion ability and charge carrier mobility of the heterostrucure. Considering that the photocatalytic process is dependent on the catalyst’s conductivity, the heterostructure interface developed between TiO_2_ and SnO_2_ semiconductors made a significant contribution. The increase of charge carriers’ mobility as well as their concentration would favor the formation of oxidative species [33] due to the additional contribution of the FTO conductive substrate (carrier mobility 13 cm^2^/Vs). The sample Ti_Al, with efficiencies of 63.8% for Tr (scenario 7) and 82.3% for Apd (scenario 7), not only benefited from the substrate conductivity but also had the additional advantage of substrate reflectivity properties allowing multi-scattering [34]. The influence of total irradiance (Figure 4c and Figure 5c) after 4 and 8 h of irradiation was more evident when UV-A contributed for more than 50% of the total irradiance. On average, in the first 4 h, 70% of the photocatalytic degradation took place for Tr and 55% for Apd, showing that the reaction kinetics depended on the pollutant molecule. In the absence of UV-A radiation, the variation of the efficiency values after 4 and 8 h was small and did not justify the use of longer periods.

The photons flux had a similar influence on Ti_FTO (Figure 4d) and Ti_Al (Figure 5d) samples. However, it is important to underline that the highest efficiency (scenario 7) was obtain when the photon flux combined both UV-A and Vis sources, meaning that the catalyst was able to benefit from both radiation sources during the generation of superoxidative species (manly ·O_2_^−^ and HO· radicals). These results confirmed that the contribution of the photon flux to the photocatalytic process was correlated to the UV share of the overall radiation sources.

Further on, the effect of the radiation properties was correlated with the kinetic data, considering the simplified Langmuir–Hinshelwood (L–H) equation (Equation (3)), usually employed to describe, overall, a photocatalytic process:

(3) $$\ln C/C_{0}=-kt$$

**Figure 6:**
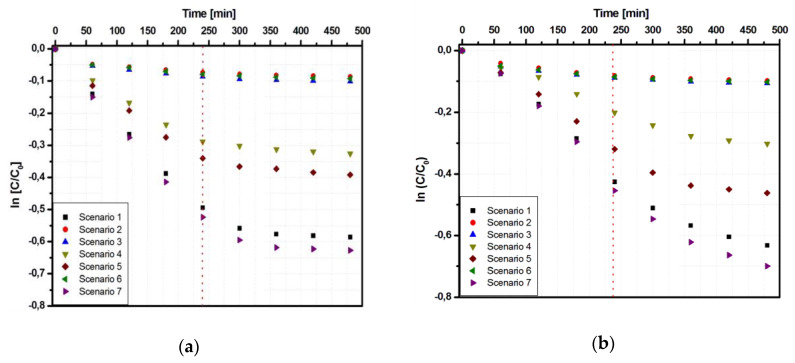
Kinetic data of the Ti_G sample using the Langmuir–Hinshelwood correlation: (**a**) Tr and (**b**) Apd photodegradation.

The kinetic evaluation of Ti_G photocatalytic properties (Figure 6) indicated the presence of two different intervals in the reaction pathway.

**Figure 7:**
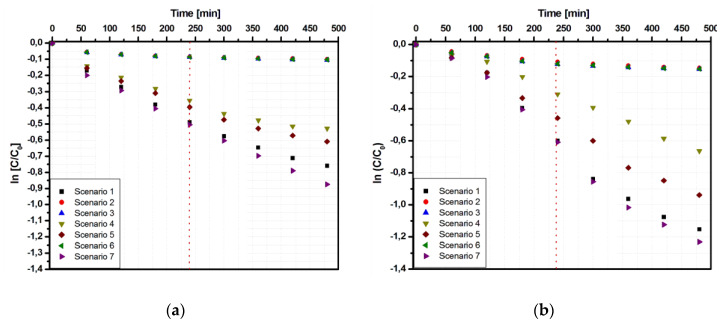
Kinetic data of the Ti_FTO sample using the Langmuir–Hinshelwood correlation: (**a**) Tr and (**b**) Apd photodegradation.

**Figure 8:**
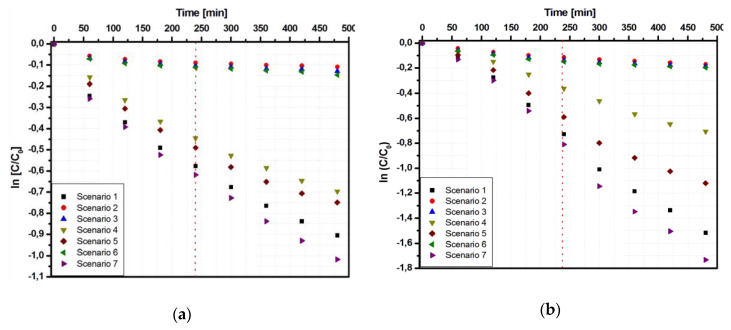
Kinetic data of the Ti_Al sample using the Langmuir–Hinshelwood correlation: (**a**) Tr and (**b**) Apd photodegradation.

The first interval (up to 240 min) corresponded to the induction period [35], followed by fast pollutant (Tr and Apd) removal [36], with a contribution of almost 75% in the total process. The second interval (from 240 to 480 min) showed a significant attenuation of the photocatalytic reaction rate, which can be attributed to surface inactivation due to the absorbance of the pollutant and the possible formation of by-products. In contrast, the samples deposed on conductive substrates such as Ti_FTO (Figure 7) and Ti_Al (Figure 8) presented a similar kinetic mechanism during the entire photocatalytic period. This result confirmed that the production of the oxidative species during the photocatalytic process was significantly influenced by the substrate nature [37].

The kinetic data model for these two-interval reaction are included in Table 3 for the Apd pollutant and Table 4 for the Tr pollutant. A closer look to the kinetic data indicates that a similar reaction rate for samples Ti_FTO and Ti_Al was measured only for the most suitable irradiation scenarios (1, 4, 5, and 7) in the case of Apd. In the other cases, the two-interval mechanism was more appropriate considering that the rate constants of the second interval were 3–4 times lower compared to those of the first interval. Another interesting finding is that the sample Ti_G exhibited a one-interval kinetic mechanism similar to Ti_FTO and Ti_Al only in the case of Apd removal using scenario 7. It was proven that scenario 7 allowed the higher photocatalytic efficiencies, but further investigations will be necessary to better understand this exception. 

The two-interval kinetic mechanism was more obvious in Tr dye removal experiments, where all parameters indicated a high reaction rate in the first interval, which was significantly reduced in the second interval. These results are consistent with an increase of dye adsorption [38] on the catalyst surface, which may block some of the active sites responsible for the production of oxidative species. Due to his chemical nature, Tr dye molecules [39] have a high affinity for the TiO_2_ surface, exhibiting an amphoteric behavior [40] as a consequence of the ionization equilibrium (OH_2_^+^, OH, O^−^).

**Figure 9:**
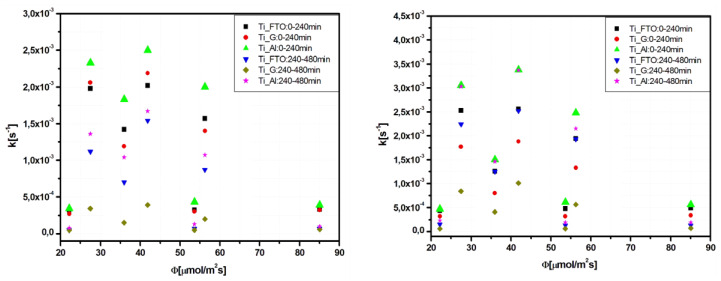
Variation of the rate constants with photon flux for (**a**) Tr and (**b**) Apd photodegradation.

The variation of these kinetic constants with the photon flux for Tr (Figure 9a) and Apd (Figure 9b) was investigated. The results indicated that the two-interval mechanism was characterized by a low reaction rate and no significant influence of the photon flux. The kinetic mechanism may be attributed to extended adsorption, which is agreement with the papers published by Somma et al. [41] and Zhao et al. [42], showing that the reduction of the photocatalytic reaction rate corresponds to the partial degradation of the pollutant, forming hydroxylated intermediates [43].

Some of these intermediates can follow the degradation pathway towards mineralization, while others may remain unchanged. However, the photon flux has a rather negligible effect on the kinetic constants in the absence of UV-A radiation. These results underline the significance of optimizing the radiation source ratio in order to influence the kinetic mechanism of photocatalytic processes.

The L–H model as proposed by Turchi and Ollis [44] was further used to model the effect of photon absorption using only scenario 7 for all samples:

(4) $$r=-\frac{dC}{dt}=\frac{k_{r}K_{S}C}{1+K_{S}C},$$

where *r* is the photocatalytic degradation rate (mol·L^−1^·min^−1^), *C* represent the TR and Apd concentration (mol·L^−1^), k_r_ is the apparent reaction rate constant (mol·L^−1^·min^−1^), and *K_s_* represent the apparent adsorption constant (L·mol^−1^). The term *k_r_·K_s_* is globally evaluated as an apparent rate constant k (min^−1^). The *k_r_* constant considers the photon flux; consequently, Equation (4) can be modified as follows:

(5) $$\frac{1}{r}=\frac{1}{k_{r}K_{S}}\times \frac{1}{C}+\frac{1}{k_{r}},$$

On a linear plot of 1/*r* vs. 1/*C*, the intercept (1/*k_r_*) and the slope (1/*k_r_K_S_*) allow calculating the kinetic parameters. The values corresponding to scenario 7 are included in Table 5.

The results indicate that the L–H equation supports the experimental data for samples Ti_FTO and Ti_Al. In these two cases, the apparent reaction rates showed the same order of magnitude of the apparent adsorption constant, while for the Ti_G sample, there was a difference corresponding to one order of magnitude. Consequently, it is feasible to conclude that when using a conductive substrate for catalyst deposition, the degradation mechanism is less affected by the radiation intensity than when using non-conductive substrates. This conclusion was also reached by Andronic et al. [45], who showed that using conductive substrates will favor the mobility of the charge carriers and the conversion to oxidative species during photocatalytic processes.

## 4. Conclusions

The influence of radiation sources, total irradiance, and photon flux on the photocatalytic removal of the dye Tr and the pesticide Apd was investigated. Titanium oxide (Degussa P25)-based films containing both anatase and rutile structures were deposed by the doctor blade technique using three types of substrate: a non-conductive substrate (microscopic glass) and two conductive (FTO and aluminum) substrates.

The presence of a conductive substrate and a suitable ratio of UV-A and Vis radiations increased the samples’ photocatalytic efficiency. The highest efficiencies were obtained for Ti_FTO (58.3% for Tr and 70.8% for Apd) and Ti_Al (63.8% for Tr and 82.3% for Apd) samples using a mixture of three UV-A and one Vis sources (13.5 W/m^2^ and 41.85 μmol/(m^2^.s)). A kinetic evaluation showed the presence of two different mechanisms of reaction, depending on irradiation time exposure and irradiation scenarios.

These results indicate that in order to optimize the design of photocatalytic processes, it is important to adjust the photon flux and the irradiation sources to the catalyst substrate and pollutant type. The use of conductive substrates compatible with the photocatalyst morphology will have a positive impact on the photocatalytic efficiency, improving the charge carrier’s mobility and the development of oxidative species. Increasing the radiation intensity can be economically non-feasible, considering that the photocatalytic processes are not characterized by a linear efficiency–irradiation evolution.

## Figures and Tables

**Table 1:** Irradiation scenarios parameters.

Irradiation Scenarios	UV-A Sources	UV-B + C Sources	Vis Sources	Total Irradiance (W/m^2^)
Scenario 1	4	0	0	12.3
Scenario 2	0	4	0	8.6
Scenario 3	0	0	4	17.6
Scenario 4	2	2	0	10.1
Scenario 5	2	0	2	14.8
Scenario 6	0	2	2	12.9
Scenario 7	3	0	1	13.5

**Table 2:** Share of UV and Vis radiation and photon flux.

Irradiation Scenarios	UV (%)		Vis (%)	Φ (μmol/(m^2^·s))
	A	B + C		
Scenario 1	100	0	0	27.46
Scenario 2	0	100	0	22.20
Scenario 3	0	0	100	85.02
Scenario 4	60	40	0	35.93
Scenario 5	40.5	0	59.5	56.24
Scenario 6	0	31	69	53.61
Scenario 7	67.5	0	32.5	41.85

**Table 3:** Kinetic data for the Apd pollutant.

Kinetic Data	Irradiation Scenarios						
	1	2	3	4	5	6	7
**Sample Ti_FTO**							
**k_0–240min_ (s^−1^)**	0.00253	0.00044	0.00049	0.00126	0.00194	0.00048	0.00256
**R^2^_0–240min_**	0.9773	0.9834	0.9792	0.9854	0.9923	0.9816	0.9841
**k_240–480min_ (s^−1^)**	0.00224	0.00015	0.00013	0.00125	0.00193	0.00013	0.00252
**R^2^_240–480_**	0.9763	0.9918	0.9895	0.9990	0.9891	0.9858	0.9816
**Sample Ti_G**							
**k_0–240min_ (s^−1^)**	0.00177	0.00032	0.00034	0.00080	0.00133	0.00032	0.00188
**R^2^_0–240min_**	0.9934	0.9576	0.9276	0.9948	0.9980	0.9295	0.9909
**k_240–480min_ (s^−1^)**	0.00084	0.00006	0.00007	0.00041	0.00056	0.00006	0.00101
**R^2^_240–480_**	0.9749	0.9863	0.9787	0.9640	0.9260	0.9796	0.9792
**Sample Ti_Al**							
**k_0–240min_ (s^−1^)**	0.00305	0.00047	0.00056	0.0015	0.00248	0.00061	0.00338
**R^2^_0–240min_**	0.9908	0.9848	0.9816	0.9955	0.9894	0.9802	0.9894
**k_240–480min_ (s^−1^)**	0.00303	0.00023	0.00019	0.00146	0.00215	0.00019	0.00337
**R^2^_240–480_**	0.9924	0.9981	0.9923	0.9942	0.9862	0.9915	0.9908

**Table 4:** Kinetic data corresponding to the Tr pollutant.

Kinetic Data	Irradiation Scenarios						
	1	2	3	4	5	6	7
**Sample Ti_FTO**							
**k_0–240min_ (s^−1^)**	0.00198	0.00032	0.00033	0.00142	0.00157	0.00032	0.00202
**R^2^_0–240min_**	0.9946	0.8915	0.8880	0.9859	0.9873	0.8897	0.9882
**k_240–480min_ (s^−1^)**	0.00112	0.00005	0.00007	0.00070	0.00087	0.00007	0.00154
**R^2^_240–480_**	0.9945	0.9981	0.9951	0.9597	0.9882	0.9914	0.9997
**Sample Ti_G**							
**k_0–240min_ (s^−1^)**	0.00206	0.00027	0.00032	0.00119	0.00140	0.00030	0.00219
**R^2^_0–240min_**	0.9988	0.8960	0.9175	0.9937	0.9949	0.9157	0.9987
**k_240–480min_ (s^−1^)**	0.00034	0.00005	0.00006	0.00015	0.00020	0.00005	0.00039
**R^2^_240–480_**	0.8609	0.9779	0.9564	0.9880	0.9647	0.9850	0.8646
**Sample Ti_Al**							
**k_0–240min_ (s^−1^)**	0.00233	0.00034	0.00039	0.00183	0.0020	0.00043	0.00250
**R^2^_0–240min_**	0.9791	0.9029	0.8964	0.9915	0.9863	0.9022	0.9808
**k_240–480min_ (s^−1^)**	0.00136	0.00008	0.00010	0.00104	0.00107	0.00013	0.00167
**R^2^_240–480_**	0.9965	0.9949	0.9917	0.9958	0.9890	0.9834	0.9985

**Table 5:** Kinetic parameters based on Equation (4) for irradiation scenario 7.

Sample, Pollutant	*k_r_*·10^8^ (mol·L^−1^·min^−1^)	*K*_s_ (mol·L^−1^)	k·(min^−1^)	R^2^
Ti_G, Tr	1.33	47243.5	0.000628	0.9386
Ti_G, Apd	1.58	54473.8	0.000838	0.9854
Ti_FTO, Tr	1.74	617562.4	0.001074	0.9942
Ti_FTO, Apd	2.81	967867.9	0.002719	0.9930
Ti_Al, Tr	2.00	709424.9	0.001418	0.9875
Ti_Al, Apd	3.82	131114.3	0.005008	0.9955
